# OPTN attenuates the neurotoxicity of abnormal Tau protein by restoring autophagy

**DOI:** 10.1038/s41398-022-02004-x

**Published:** 2022-06-04

**Authors:** Yin Xu, Yun Liu, Xi Chen, Qia Xu, Liwei Liu, Hui Liu, Ruowen Guo, Yide Qin

**Affiliations:** 1 School of Basic Medical Sciences, Anhui Medical University, 230032 Hefei, Anhui P.R. China; 2 The Second Affiliated Hospital of Anhui Medical University, 230601 Hefei, Anhui P.R. China; 3 Laboratory of Molecular Neuropsychology, School of Mental Health and Psychological Sciences, Anhui Medical University, 230032 Hefei, Anhui P.R. China; 4 Centers of Disease Control and Prevention, Xinfeng County, Shaoguan, 511100 Guangdong, P.R. China

**Keywords:** Molecular neuroscience, Diseases

## Abstract

OPTN is an autophagy receptor involved in autophagic degradation. Here we studied the role of OPTN in attenuating the neurotoxicity induced by mutated Tau protein. We constructed recombinant adeno-associated viruses with OPTN and Tau-P301L genes, respectively. Through virus coinfection on neuronal cell line HT22 in vitro and Kunming mice in vivo, we found that autophagy- and apoptosis-associated genes are altered by Tau-P301L at both mRNA and protein levels, which are restored by OPTN expression. Functionally, OPTN suppresses apoptosis and enhances cellular viability in Tau-P301L expressing HT22 cells, and increases learning and memory in Tau-P301L expressing mice, respectively. Last, we found that OPTN reduces the p-Tau levels in vitro and in vivo. Our results reveal the function of OPTN in lowering the p-Tau level and the expressions of apoptosis genes, and increasing the expressions of autophagic genes, indicating a beneficial role of OPTN in Tau pathology.

## Introduction

Alzheimer’s disease (AD), the most common neurodegenerative disorder in the aged population, is characterized by cognitive decline and memory loss [1, 2], which affects more than 50 million people all over the world [3]. Neurofibrillary tangle (NFT) caused by abnormally phosphorylated Tau proteins (encoded by *MAPT* gene) is one of the classic pathological hallmarks of AD [4, 5]. The *MAPT* gene locates at the 17q21 site of the chromosome and contains 16 exons. Through alternative splicing, six isoforms of Tau proteins can be translated into the brain [6]. *MAPT* gene mutations lead to Tau hyperphosphorylation (p-Tau) and NFT deposition in the brains and cause genetic forms of neurodegenerative diseases [7]. Therefore, lowering mutant Tau could be a potential therapeutic strategy for reducing the Tau hyperphosphorylation and NFT deposition. However, over the past decades, only a few clinical trials have limited progress on AD, which urges the development of novel effective therapeutic approaches.

Autophagy, a highly conserved bulk degradation process within eukaryotic cells, is responsible for cellular materials turnover and nutrient self-supply during starvation [8], which promotes cells survival in harsh environments. The substrates of autophagy include protein aggregates, invading pathogens, and damaged organelles. In pathological conditions, autophagy also serves as a defense mechanism to degrade pathological proteins such as p-Tau.

Optineurin (OPTN) is a crucial autophagy receptor that facilitates the recognition of infected pathogens, protein aggregates, and mitochondria [9, 10]. The depletion of OPTN significantly increases the formation of protein aggregates and results in a loss of function of selective autophagy [11]. In contrast, upregulating the expression of OPTN can induce autophagy [12]. As an autophagy receptor, OPTN grants autophagy a precise cargo selection, where only specific substrates are engulfed within autophagosomes and delivered to the lysosome for proteolytic breakdown [8]. However, it is not fully understood whether OPTN affects autophagy in the presence of mutant Tau and how it affects mutant Tau accumulation.

Here, we investigated the role of OPTN in the clearance of abnormal Tau protein and its regulation of autophagy and apoptosis. We found that OPTN induces the clearance of abnormal Tau protein, enhances autophagy, and downregulates caspase-dependent apoptosis. Meanwhile, overexpression of OPTN significantly reduced pathological Tau in mice expressing Tau-P301L. Our findings show a beneficial role of OPTN in the presence of mutant Tau.

## Materials and methods

### Reagents

Trizol was purchased from Thermo Fisher, USA. A reverse transcription kit was purchased from Takara Company. Fetal bovine serum was purchased from Kangyuan Biotechnology Co., Ltd. Pancreatin and RIPA lysate were purchased from Biyuntian Biotechnology Co., Ltd. DMEM and Lipofiter™ 3000 were, respectively, purchased from Hyclone, USA and Invitrogen, USA. Opti-mum Cutting Temperature Compound (OCT) was purchased from SAKURA, USA. Rabbit polyclonal or monoclonal antibodies of optineurin (OPTN) (catalog #: ab151240), Tau (catalog #: ab76128), p-Tau (phospho S404) (catalog #: ab92676), LC3 (catalog #: ab48394), Beclin1 (catalog #: ab207612), Caspase-3 (catalog #: ab4051), cleaved caspase‑3 (catalog #: ab49822), and β-actin (catalog #: ab8227) were purchased from Abcam, USA. And horseradish peroxidase-conjugated secondary antibody (goat anti-mouse or goat anti-rabbit IgG) was purchased from Pierce, USA.

### Cell line and plasmids

Human embryonic kidney (HEK) cell line 293 T, mouse hippocampal neuronal cell line HT22, and *Escherichia coli* Top 10 were originally purchased from American Type Culture Collection, America (ATCC) and were preserved by the Biochemistry and Molecular Biology Laboratory of Anhui Medical University. The plasmids of adeno-associated virus (AAV): AAV-Green with green fluorescent protein gene, AAV-Red with red fluorescent protein gene, AAV-RC9, and pHelper were purchased from Shanghai Novobio Scientific Co., Ltd, China. The serotype and promoter of AAVs are AAV9 and CMV, respectively. pUC57 plasmid was preserved by the Biochemistry and Molecular Biology Laboratory of Anhui Medical University. 293 T and HT22 cells were cultured with DMEM containing 10% FBS (from Gibco), 80U/ml streptomycin, and 100U/ml penicillin at 37 °C in 5% CO_2_ and passaged by 0.4% trypsin to maintain logarithm growth for the subsequent experiment.

### Construction and verification of recombinant AAV plasmids

The primers for cloning *OPTN* and *MAPT* genes were designed according to the NCBI gene sequence (*OPTN*, NM_001008211.1; *MAPT*, NM_016834.5), and the target genes were synthesized by Sangon Biotech Co., Ltd. (Shanghai, China). The cleavage sites of restriction enzymes *EcoR I* and *BamH I* were added to the forward and reverse primers, respectively. The pUC57 plasmids carrying the *OPTN* and *MAPT* genes were used as the templates to amplify the *OPTN* and *MAPT* genes through PCR. The forward (F) and reversed (R) primers of OPTN were 5′-GCCTCCGCGGATTCGAAATGTCCCATCAACCTCTCAGCT-3′ and 5′-TTCAATCGATGTTCGAATTAAATGATGCAATCCATCACGTGAATCTG-3′, respectively. The forward (F) and reversed (R) primers of *MAPT* were 5′-TATGGCCACAGGATCCATGGCTGAGCCCCGC-3′ and 5′-TCATCGATATGGATCCTCACGTAGAATCGAGACCGAGGAG-3′, respectively. The plasmids (AAV-Green and AAV-Red) and the amplified genes (*OPTN* and *MAPT-P301L*) were digested with both *EcoR I* and *BamH I*, respectively, and were then ligated through a standard T4 ligase. Then, the AAV-Green-*OPTN* and AAV-Red-*MAPT*-P301L plasmids were transformed into *E. coli* TOP10 for bacterial culture. The positive clones were selected, and the recombinant plasmids were purified, further identified by restriction endonucleases, and sequencing. The sequencing results were validated with the NCBI queries.

### Package and titer of AAVs

Recombinant AAVs were produced by cotransfecting 293 T cells with constructed plasmids (AAV-Green-*OPTN* or AAV-Red-*MAPT*-P301L), envelope plasmid AAV-RC9 and assistant plasmid pHelper using Lipofectamine. The detailed protocol was reported previously [13]. 293 T cells were seeded into wells of six-well plates at a density of 4 × 10^5^ cells/well for culturing for 72 h in DMEM with 10% FBS, the culture medium was replaced with the fresh one after 24 and 48 h incubation. All the cells were collected, lysed by freeze-thaw, and centrifuged at 10,000 × *g* for 30 min at 4 °C, and the supernatants rich in AAV virus particles were collected. The virus solution was purified with iodixanol gradient ultracentrifugation and concentrated with PEG8000 [14]. Then, the titers were measured by real-time quantitative PCR (qPCR). The F and R primers of qPCR were 5′-GAGTGGCCAACTCCATCACT-3′ and 5′-CGTTACTATGGGAACATACGT-3′, respectively. The reaction conditions were as follows: 95 °C pre-denaturation 180 s; 94 °C × 30 s, 62 °C × 30 s, 72 °C × 30 s (40 cycles).

### Proliferation and apoptosis analysis of AAV infected HT22 cells

The HT22 cells were maintained in the logarithmic growth stage. The cells were seeded into 96-well plates in sextuplicate at a density of 5 × 10^3^ cells/well. After 24 h, the cells were infected with AAV at 10^6^ multiplicity of infection (MOI) (MOI = virus number/cell number) including AAV-Green-*OPTN* and/or AAV-Red-*MAPT*-P301L in serum-free DMEM for 10 h, and with complete medium for 48, 72, and 96 h, respectively. The experiments were divided into six groups: control, Green control, Red control, OPTN, Tau-P301L, and OPTN + Tau-P301L groups, which were infected AAV-Green, AAV-Red, AAV-Green-*OPTN*, AAV-Red-*MAPT*-P301L, and AAV-Green-*OPTN* + AAV-Red-*MAPT*-P301L viruses, respectively, except for the control group.

The proliferation of the cells was measured by the MTT [3-(4,5-dimethylthiazol-2-yl)-2,5-diphenyltetrazolium bromide] assay, as described previously [15]. The following formula was used to calculate the cell viability (%) = (experimental group A_490nm_/control group A_490nm_)×100%. Each experiment was sextuplicated in two independent sets.

Apoptosis of HT22 cells at 72 h time point was measured by flow cytometry (EPICSRXL-MCL, Beckman, USA) using FITC-conjugated annexin-V and propidium iodide (PI) from Sigma. The experimental operating procedure followed the manufacturer’s manual. The results of flow cytometry were analyzed using FlowJo v10.6.2 (USA). Cells that stained positive for annexin-V were counted as apoptotic cells. The apoptosis rate means early apoptosis (Q2 of flow cytometry dot plot) and late apoptosis (Q3 of flow cytometry dot plot). The experiment was performed with triplicate in two independent sets.

### Model of animals infected with AAV

Three healthy male and 20 female Kunming mice weighing 20–26 g were purchased from Anhui Medical Experimental Animal Center (No.0014983). All animal experiments were performed according to the experimental program (LLSC20180132) approved by The Experimental Animal Ethics Committee of Anhui Medical University. All methods and experimental protocols reported in this paper for related studies, including animal use, cell culture, and in vivo, studies were performed following the guidelines and regulations of the committee’s protocol. All mice were kept in SPF-class sterile room at (22 ± 2) °C, (40–60)% relative humidity, and a 12 h light/dark cycle in the Anhui Provincial Center for Medical Experimental Animals with free access to food and drinking water. The mice's suffering was ameliorated as much as possible during animal experiments. The mice were sacrificed one day after the last behavioral test. All mice were euthanized at the end of the experiments.

Maternal mice were fed with high fat during pregnancy (A high-fat diet in mice during pregnancy prevents Alzheimer’s disease in the offspring) [16]. 40 Kunming P0 mice (born within 24 h) were randomly divided into four groups (*n* = 10/group): control, OPTN, Tau-P301L, and OPTN + Tau-P301L, which were, respectively, injected saline, AAV-*OPTN*, AAV-*MAPT*-P301L, and AAV-*OPTN* + AAV-*MAPT*-P301L viruses in the bilateral ventricles (2/5 of the distance from the λ sutures to each eye) at 2 μl/chamber (10^12^vg/ml) containing 0.05% Trypan Blue dye. Both mouse genders were equally used in each group. The specific operation was as follows. The P0 mice were transferred to an ice metal plate covered with ice for 5 min, then wiped the head with 75% alcohol. The microinjector was held in the mouse’s head at about 30° (with the needle toward the cerebellum) and injected into the mouse's head at about 4 μm. The microinjector was slowly pushed to inject 2 μl and waited for the virus to spread for 10 s, as described in the previous report [17]. If the virus was injected into the right place, the virus could spread throughout the brain that was seen in blue. If the virus entered the thalamus, its transmission was limited and the brain was not seen in blue. After injecting the left and right hemispheres of the mice, the mice were put back in the cotton wool below 50–70 °C water until their body temperature and skin tone returned to normal and they began to move, returning the mice to their biological mothers and making sure they smelled like mice. All the virus-related experiments were carried out in a biosafety cabinet to prevent biological contamination. The mice were isolated and reared in an SPF-class sterile room for 72 h. Mice were fed for 4 months for follow-up experiments.

Investigators were blinded to group identities during data collection and analysis.

### Behavior tests

The mice in four groups (*n* = 10 / group) at 4 months were performed passive avoidance experiments to observe their learning and memory changes. The animals were tested for their learning and memory in an apparatus consisting of two inter-connected light and dark chambers (each: 20.3 × 15.9 × 21.3 cm), in which the illumination indices of the dark and light chamber were 300 lux and less than 1 lux, respectively, and the dark chamber was equipped with an electric grid floor. The mice were trained to adapt to the environment on the first day. On the second day, the passive avoidance chamber was electrified, and the mice were taken out after the shock, in which the intensity of shock was 0.3 mA (100 V, 50 Hz AC) for 2 s. On the third day, the formal experiment began. The passive avoidance chamber was electrified, then the number of mice entering the dark chamber in 5 min (number of errors) and times of mice first entering the dark chamber (latent period) were recorded after the mice were placed in the lighted chamber. If the mouse still did not enter the dark chamber for 5 min, the number of errors was recorded as 0 times and the latency period as 300 s.

### Reverse transcription‑quantitative PCR (RT‑qPCR)

At the endpoint of the experiment, the cells and mice brain tissues were harvested, in which the total RNAs were extracted with TRIzol® reagent according to the manufacturer’s instructions. cDNA was synthesized in a reverse transcription reaction system. The reverse transcription reaction conditions were 42 °C for 15 min and 95 °C for 5 min.

The primers were designed by Primer 6.0 software according to genes’ sequences searched by Primer-Bank, and synthesized by Shanghai Shenggong Biological Co., Ltd. (Supplementary Table 1). The mRNA expression levels of *Map1lc3a* (LC3), *Becn1* (Beclin1*)*, and *Casp3* (*Caspase-3*) in HT22 cells or mice brain tissues were measured in ABI7500 fluorescent real-time PCR instrument (Applied Biosystems; Thermo Fisher Scientific, Inc.). Real-time PCR adopts TaKaRa SYBR Green as real-time PCR Master Mix. *Actb* (β-actin) was used as the housekeeping gene. The reaction conditions were as follows: 95 °C pre-denaturation 300 s; 95 °C × 20 s, 56 °C × 30 s, 72 °C × 30 s (40 cycles). The critical threshold (C) was determined using ABI SDS software (Applied Biosystems), which was defined as the number of cycles in which the linear phase of each sample exceeded the threshold level. For each target gene, mRNA expression levels were calculated using the 2^−ΔΔCq^ method (ΔCq = target gene Cq − housekeeping gene Cq value) [18]. All reactions were performed in triplicate, and a mixture lacking a complementary DNA template was used as the negative control. Two independent experiments were run.

### Western blot

The cells and mice brain tissues collected and harvested were lysed using RIPA buffer with 1% phenylmethanesulfonyl fluoride (PMSF). The protein concentration was determined by the BCA method. Proteins isolated from cells or mice brain tissues were separated by SDS‑PAGE [5% stacking gel, 10 or 12% lower gel (w/v); 22.5 μg protein in 15 μl loaded per lane], and subsequently transferred to PVDF membranes. After blocking with 5% milk, the membranes were incubated with primary antibodies (1:1000) and then incubated with horseradish peroxidase-conjugated secondary antibody (HRP‑conjugated goat anti‑rabbit IgG, 1:8000) overnight. Proteins were exposed and detected by ECL (Electro-Chemi-Luminescence) system in a chemiluminescent imaging system (Clinx Science Instruments Co., Ltd.), and intensities were quantified using Quantity-One software version 4.62 (Bio-Rad). The β-actin was used as a loading control. All reactions were performed in triplicate, and two independent experiments were performed.

### Immunofluorescence and immunohistochemistry

The whole mice brain was removed from the skull and fixed in 4% paraformaldehyde in PBS at 4 °C for 24 h. The fixed brain was transferred to a 30% sucrose solution, which was incubated at 4 °C until the brain sank to the bottom. After OCT (optimal cutting temperature compound) embedding, the tissue was sectioned in a freeze slicer with a thickness of 30 μm. The cut tissue was transferred to a PBS solution and then attached to an anti-detachment slide, which was observed under an upright fluorescence microscope and photographed at ×100 magnification.

Mounted paraffin sections of 4 μm thickness were de-waxed with xylene and dehydrated with ethanol. After antigen retrieval with high pressure and block of endogenous peroxidase with 3% H_2_O_2_, the sections are incubated for 40 h at 4 °C with the rabbit polyclonal antibody of OPTN and the rabbit monoclonal antibody of p-Tau, respectively, (1:1,000, Abcam) and thereafter processed for 1 h with horseradish peroxidase-conjugated secondary antibody (goat anti-rabbit IgG, 1:8000, Pierce). Reactions were visualized with the DAB color developer (Pierce). ImageJ software (version 1.44; National Institutes of Health) was used for quantitative colocalization analysis.

### Statistical analysis

The data was analyzed by SPSS 20.0 (IBM Corp.). The results are expressed as mean ± standard deviation (SD). The comparisons were analyzed with one-way ANOVA followed by Tukey’s post-hoc test. *P* < 0.05 was the standard of statistical difference.

## Results

### Construction of AAV containing *OPTN* or *MAPT*-P301L

The recombinant plasmids (AAV-Green-*OPTN* and AAV-Red-*MAPT*-P301L) were constructed, in which the sequencing results of the target genes are shown in Supplementary Fig. 1. The AAVs were produced by cotransfecting 293 T cells with constructed recombinant plasmids, envelope plasmid AAV-RC9, and assistant plasmid pHelper using Lipofectamine. The green fluorescent protein (GFP) or red fluorescent protein (RFP) expressions were observed by a fluorescence microscope (Supplementary Fig. 2). By qPCR analysis, the viral titer was 1.5 × 10^12^ vg/ml (Supplementary Table 2 and Supplementary Fig. 3).

### AAV-*OPTN* enhanced the viability of neuronal cell line infected with AAV-*MAPT*-P301L and reduced its apoptosis in vitro

**Fig. 1:**
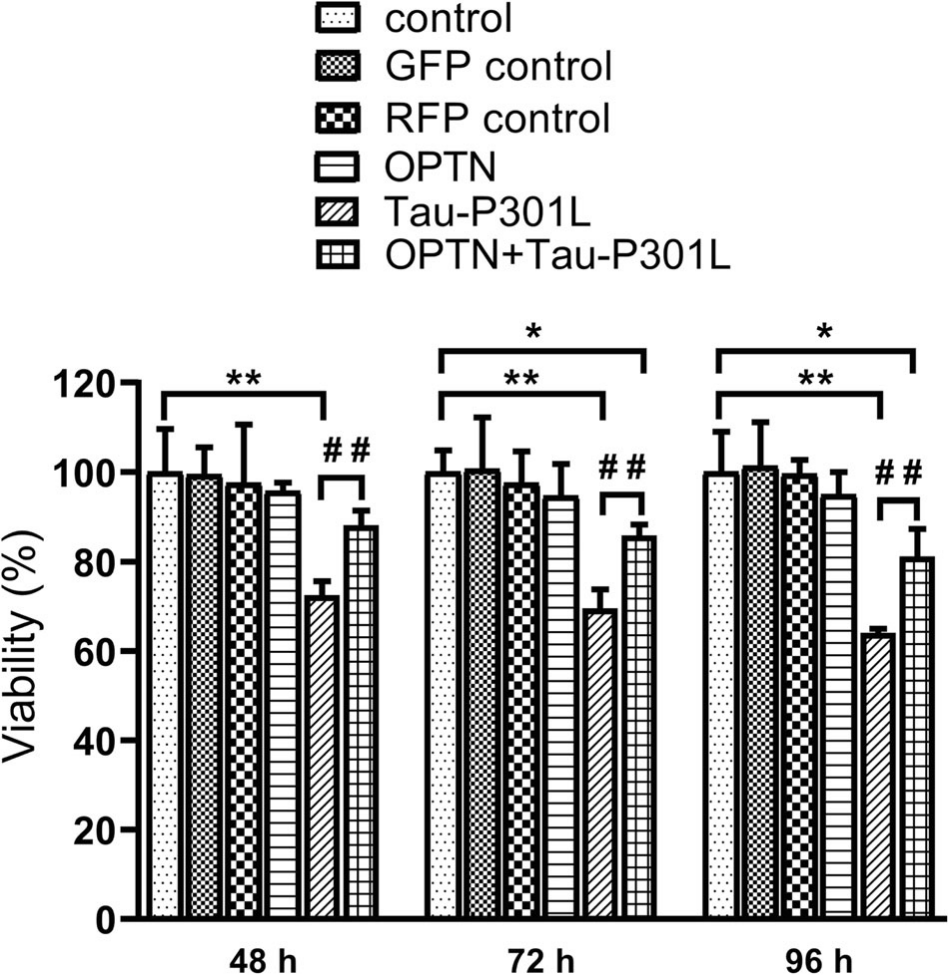
AAV-*OPTN* enhanced the viability of infected with AAV-*MAPT*-P301L in vitro. Data are presented as mean ± SD, the experiment was performed with sextuplicate in two independent sets, *P < 0.05, **P < 0.01 compared with control; ^##^*P* < 0.01 compared with TAU-P301L. GFP green fluorescent protein, RFP red fluorescent protein.

We used HT22 cells as a research model in vitro. Compared to control, the cell viability was significantly suppressed after Tau-P301L overexpression, which was rescued by OPTN overexpression, in which showed a time-dependent manner (Fig. 1).

**Fig. 2:**
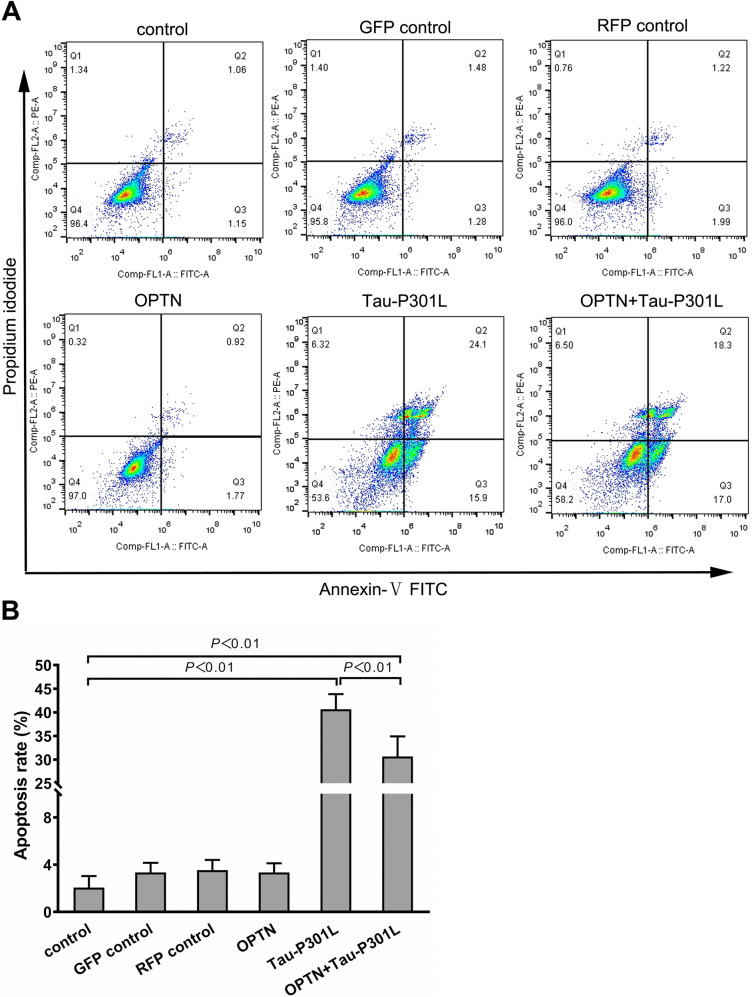
AAV-*OPTN* reduced the apoptosis of neuronal cell line HT22 infected with AAV-*MAPT*-P301L for 72 h in vitro. **A** Representative flow cytometry dot plot of neuronal cell HT22 line stained with annexin-V-FITC and PI. **B** Histogram of apoptosis rates of neuro cell line. The data are shown as means ± SD, the experiment was performed with triplicate in two independent sets. GFP green fluorescent protein, RFP red fluorescent protein.

Compared to control, the apoptosis of cells expressing Tau-P301L significantly increased at 72 h after infection, which was reduced by OPTN expression (Fig. 2). These results show a beneficial role of OPTN in Tau-P301L expressing neuronal cell lines.

### Mouse brain was infected with AAV carrying *OPTN* or/and *MAPT*-P301L

**Fig. 3:**
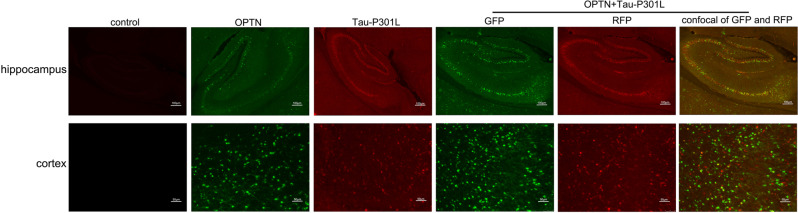
The brain tissue from 4-months-old mice injected with AAV at P0 phase were visualized by fluorescent microscopy. GFP green fluorescent protein, RFP red fluorescent protein.

By fluorescent imaging, OPTN protein and Tau protein achieved high colocalization in the brains of 4-months-old mice, specifically in the cortex and hippocampus, which are critical brain regions in learning and memory. (Fig. 3). This result indicates that OPTN protein targets the mutate Tau protein.

### OPTN alleviates the decline of learning and memory in mice expressing Tau-P301L

**Fig. 4:**
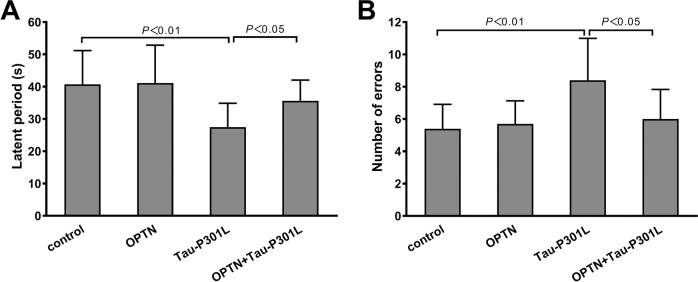
OPTN redressed the depression of mice in learning and memory (passive avoidance) abilities induced by AAV-*MAPT*-P301L. **A** Time of mice first entering the dark chamber (latent period). **B** Number of mice entering the dark chamber in 5 min (number of errors). The data are shown as means ± SD, *n* = 10.

The passive avoidance experimental results showed that latent periods in mice infected with AAV-*MAPT*-P301L were significantly shorter than that of the control group. meanwhile, the number of errors significantly increased (Fig. 4). OPTN expression significantly mitigated the impact of AAV-*MAPT*-P301L. The latent periods in the OPTN + Tau-P301L group were significantly prolonged, and the number of errors significantly decreased, which were close to control levels (Fig. 4). The aforementioned two indicators in the OPTN group were similar to the control group (Fig. 4). These results suggest that OPTN reduces the decline in learning and memory in mice caused by mutant Tau protein.

### OPTN regulates the mRNAs of associated genes in cells and mice brain tissues infected with AAV

**Fig. 5:**
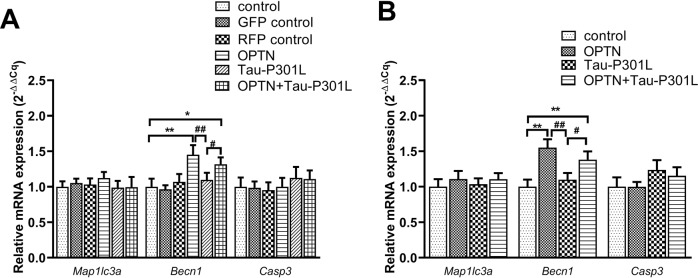
OPTN regulates the mRNA level of autophagy- and apoptosis-associated genes in cells and mice brain tissues infected AAV. *Actb* was used as the reference gene. **A** Neuronal cell line HT22. **B** The brain tissue of Kunming mice. Data are presented as the mean ± SD (cell line: *n* = 6; mice: *n* = 10). *P < 0.05, **P < 0.01 compared with control; ^#^*P* < 0.05, ^##^*P* < 0.01 compared with Tau-P301L^.^ GFP green fluorescent protein, RFP red fluorescent protein.

To study the underlying mechanism of OPTN affecting mutant Tau and relieving its neurotoxicity, we tested the mRNA expression of autophagic and apoptosis genes by real-time PCR. In neuronal cell line HT22, as expected, *Becn1* mRNA was increased in the AAV-*OPTN* group, but *Casp3* and *Map1lc3a* mRNAs remained unchanged compared to the control cells (Fig. 5A). Neither *Becn1*, *Casp3* nor *Map1lc3a* mRNA levels in Tau-P301L group cells treated with AAV-*MAPT*-P301L had changed much (Fig. 5A). In AAV-*OPTN* and AAV-*MAPT*-P301L co-treatment group, OPTN significantly increased Becn1 expression compared to AAV-MAPT-P301L group (Fig. 5A). However, the aforementioned changes were not observed between the control group and the GFP control/RFP control groups (Fig. 5A).

In the mice infected with AAV, *Becn1* mRNA in the OPTN group was significantly increased, while *Casp3* and *Map1lc3a* mRNAs remained unchanged compared with the control (Fig. 5B). Neither *Becn1*, *Casp3* nor *Map1lc3a* mRNA levels in Tau-P301L group mice treated with AAV-*MAPT*-P301L had changed much (Fig. 5B). In AAV-*OPTN* and AAV-*MAPT*-P301L co-treatment group, the mRNA of *Becn1* exhibited a significant increase, and *Map1lc3a* and *Casp3* mRNAs remained unchanged (Fig. 5B). Compared to the Tau-P301L group, the mRNA level of *Becn1* of the AAV-*OPTN* and AAV-*MAPT*-P301L co-treatment groups was significantly increased, and *Casp3* mRNA level had a downward trend but was not statistically significant (Fig. 5B).

### OPTN regulates the levels of proteins associated with autophagy and apoptosis in cells and mouse brains expressing Tau-P301L

**Fig. 6:**
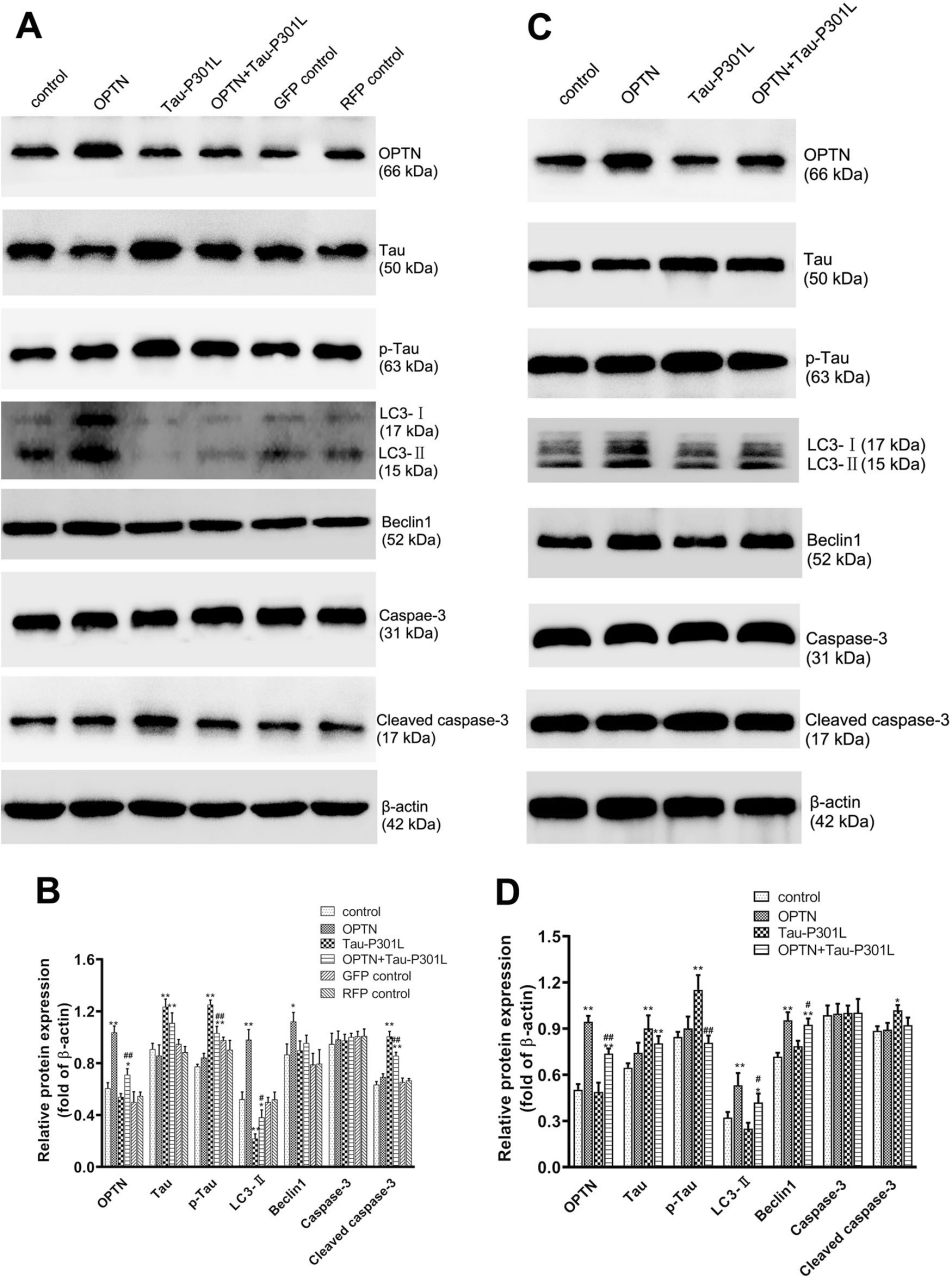
OPTN regulates the levels of proteins associated to autophagy and apoptosis in cells or mice brain tissues infected AAV. **A** The related proteins were detected with western blotting in neuronal cell line HT22, and β-actin was used to show the similar amount of protein loaded in different lanes. **C** The related proteins were detected with western blotting in the brain tissues of mice, and β-actin was used as the reference protein. **B**, **D** Relative intensities of protein bands in **A** and **C** were determined using Quantity-One software and normalized to β-actin band intensity. Data in **B** and **D** are presented as mean ± SD (cell line: *n* = 6; mice: *n* = 10). *P < 0.05, **P < 0.01 compared with control; ^#^*P* < 0.05, ^##^*P* < 0.01 compared with TAU-P301L^.^ GFP green fluorescent protein, RFP red fluorescent protein.

The protein expression levels of OPTN, Tau, p-Tau, LC3I/II, Beclin1, Caspase-3, and Cleaved caspase-3 were assayed by western blotting. In HT22 cells, the expressions of OPTN, LC3II, and Beclin1 proteins in the OPTN group were significantly increased, while the expressions of Tau, p-Tau, Caspase-3, and Cleaved caspase-3 proteins are similar, compared to the control group (Fig. 6A, B). The expressions of Tau, p-Tau, Caspase-3, and Cleaved caspase-3 proteins increased significantly, while OPTN, LC3I/II, and Beclin1 proteins remained unchanged in the Tau-P301L group (Fig. 6A). In the group treated with AAV-*OPTN* + AAV-*MAPT*-P301L, the same changes observed in Tau-P301L group became less significant (Fig. 6A). However, these changes were not significant between the control group and the GFP control/RFP control groups (Fig. 6A). This result suggests that OPTN induces autophagy responses and reduces apoptosis caused by p-Tau.

In Kunming mice infected with AAVs, the changes in autophagy and apoptosis-associated proteins were similar to that in HT22 cells in vitro. Compared to the control group, in the OPTN group, OPTN, Beclin1, and LC3-II protein levels were significantly increased, and compared to Tau P301L group, p-Tau level was significantly decreased in OPTN + Tau P301L group, while no significant changes were observed on the levels of Tau, Caspase-3, and Cleaved caspase-3 (Fig. 6C, D). Compared with the Tau-P301L group, these changes were blunt in the OPTN + Tau-P301L group, which showed that OPTN reduced the effect of the p-Tau on the mouse brain, increasing autophagy.

**Fig. 7:**
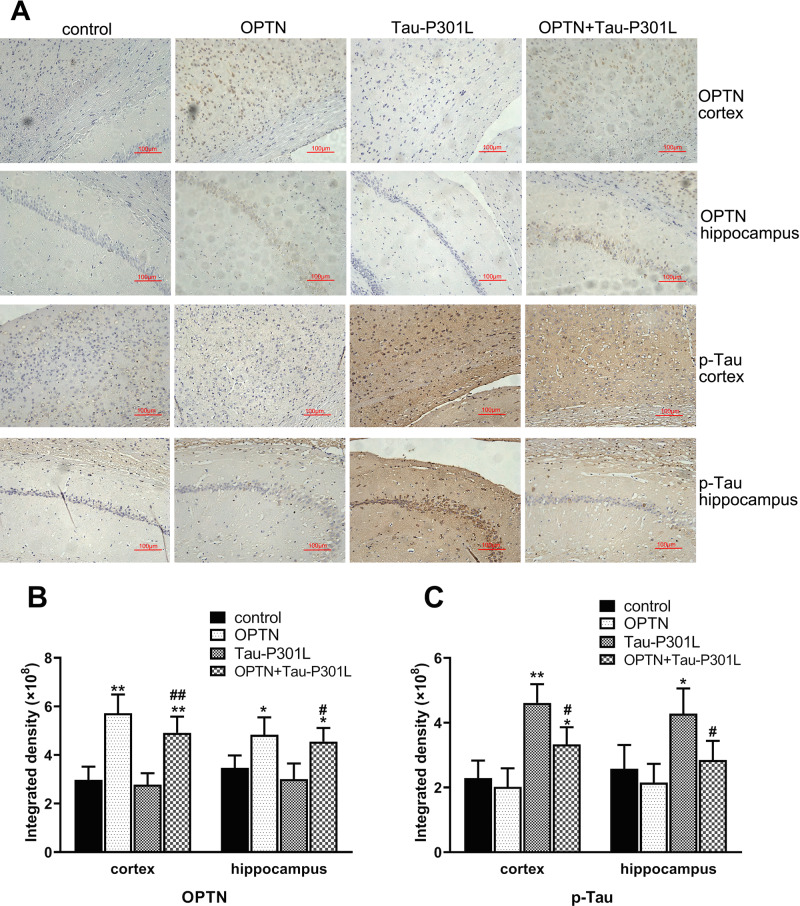
Immunohistochemical analysis of OPTN and p-Tau in brain tissues of 4-months-old mice infected with AAV in stage P0. **A** Immunohistochemical sections of OPTN and p-TAU in the cortex and hippocampus of mouse brain tissues (×200). **B** Histogram of OPTN expression of the cortex and hippocampus. **C** Histogram of p-Tau expression of the cortex and hippocampus. The data are shown as means ± SD, *n* = 10. *P < 0.05, **P < 0.01 compared with control; ^#^*P* < 0.05, ^##^*P* < 0.01 compared with Tau-P301L.

By immunohistochemical experiments, we analyzed the expressions of OPTN and p-Tau in the cortex and hippocampus of Kunming mice injected AAVs 4 months after P0 injection. As expected, the protein level of OPTN in the AAV-*OPTN* group significantly increased compared with the control group, also in the AAV-*OPTN* and AAV-*MAPT-P301L* co-treated group (Fig. 7). Compared to the control group, p-Tau in the Tau-P301L group significantly increased, while OPTN remained unchanged (Fig. 7). Compared with the Tau-P301L group, p-Tau change significantly lessened in the AAV-*OPTN* and AAV-*MAPT*-P301L co-treated group (Fig. 7).

## Discussion

Adeno-associated viruses, as the most common shuttle vectors, have exhibited the ability to deliver target genes safely and effectively. In this experiment, we used adeno-associated viruses to specifically infect neuronal cell line HT22 and Kunming mice. The brains of mice were not mature within the first 0–24 h after birth (P0 mice). The injection of AAV in the ventricle in P0 mice led to extensive neuronal transduction in the whole brain [19, 20]. The gene expression begins within a few days after injection and lasts for a lifetime.

**Fig. 8:**
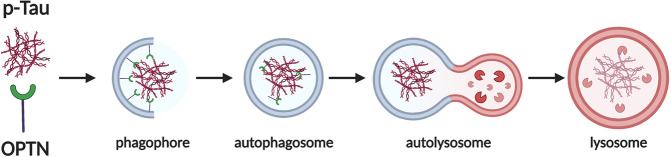
Diagrammatic sketch of the mechanism of OPTN. OPTN as an autophagy receptor recognizes p-tau and facilitates its autophagic degradation. Created with BioRender.com.

Multiple studies have shown that the treatment targeting pathological Tau is a promising direction for Alzheimer’s disease research and OPTN has great potential in clearing pathological Tau and alleviating neurotoxicity [21, 22]. The abnormally p-Tau protein is a major component of NFT, which is associated with cognitive dysfunction in AD [23]. The C terminal of OPTN protein contains the LC3-interacting region (LIR) and ubiquitin-binding area (UBA) [24, 25]. The autophagy receptor OPTN, besides the UBA and LIR domains, also has some interacting domains of KIR, TB, ZZ, and PB1 proteins. By interacting with the LC3 and ubiquitin-conjugated substrates, OPTN bridges the autophagy with the substrates and pulls them into autophagosomes, thereby achieving selective autophagy [26] (Fig. 8). OPTN plays a key role in triggering autophagy and initiating cell response to stress through protein interactions. OPTN may isolate the free toxic and apoptotic proteins in the cytoplasm by interacting with protein aggregates [8, 26, 27]. OPTN has multiple functional domains, which determines that it may participate in many signal pathways, but the specific mechanism of action is not still unclear. In this study, our results indicated that OPTN, as a selective autophagy receptor, may promote the elimination of abnormal Tau protein to relieve neurotoxicity via enhancing autophagy. We found that overexpression of OPTN enhanced autophagy and downregulated the phosphorylation of Tau protein both in vivo and in vitro.

LC3 is one of the most important participants of autophagy, which is required for the autophagic process [28]. A recent study showed that OPTN is sufficient to recruit LC3 to damaged mitochondria and induce mitochondrial autophagy [29]. LC3 can be used for detecting the activity of autophagy. Beclin1 is a key molecule that regulates cell autophagy. Beclin1 is phosphorylated by ULK1 (unc-51 like autophagy activating kinase 1), then it activates lipase VPS34 (vacuolar protein sorting 34) to induce cell autophagy [30, 31]. Beclin1 has a BH3 domain, and Bcl-2 can affect its activity by binding the BH3 domain of Beclin1. The relative number of Bcl-2 and Beclin1 binding to each other in the cell largely determines the autophagy level of the cell. Thus, cellular autophagy will be expected to increase by interfering Bcl-2 binding to Beclin1 or upregulating Beclin1 expression level [30]. We found that overexpression of OPTN promoted the expression level of Beclin1 and the transformation of LC3-I to LC3-II. This suggested that OPTN could activate autophagy consistency with previous studies.

We demonstrated that OPTN efficiently downregulate the cell apoptosis caused by abnormal Tau protein. Caspase-3 is a kind of executioner caspase that plays an essential role in apoptosis [32]. Our results showed that the activated Caspase-3 (Cleaved caspase-3) level significantly increased in the Tau-P301L group, while Cleaved caspase-3 in the OPTN + TAU-P301L group significantly decreased.

Our data suggest that OPTN eliminated abnormal Tau protein by regulating autophagy. However, the exact molecular mechanism of OPTN in removing abnormally Tau protein needs further study in the future. Furthermore, the detailed mechanism of the interaction between apoptosis and autophagy in Alzheimer’s disease is still not fully understood. However, virus-mediated overexpression of OPTN in the treatment of Alzheimer’s disease has a promising application. In the present study, we provided proof for the specific role of OPTN in the clearance of hyperphosphorylation Tau protein, which may provide a possible avenue in the treatment of Alzheimer’s disease.

## Supplementary information

Supplementary Figure 1

Supplementary Figure 2

Supplementary Figure 3

Supplementary Table 1

Supplementary Table 2

## Data Availability

The datasets used and/or analyzed during the current study are available from the corresponding author on reasonable request.
