# Supplementary figures and images for: OPTN attenuates the neurotoxicity of abnormal Tau protein by restoring autophagy
Source: Transl Psychiatry. 2022 Jun 4;12:230. doi: 10.1038/s41398-022-02004-x (PMC9167278; doi:10.1038/s41398-022-02004-x)

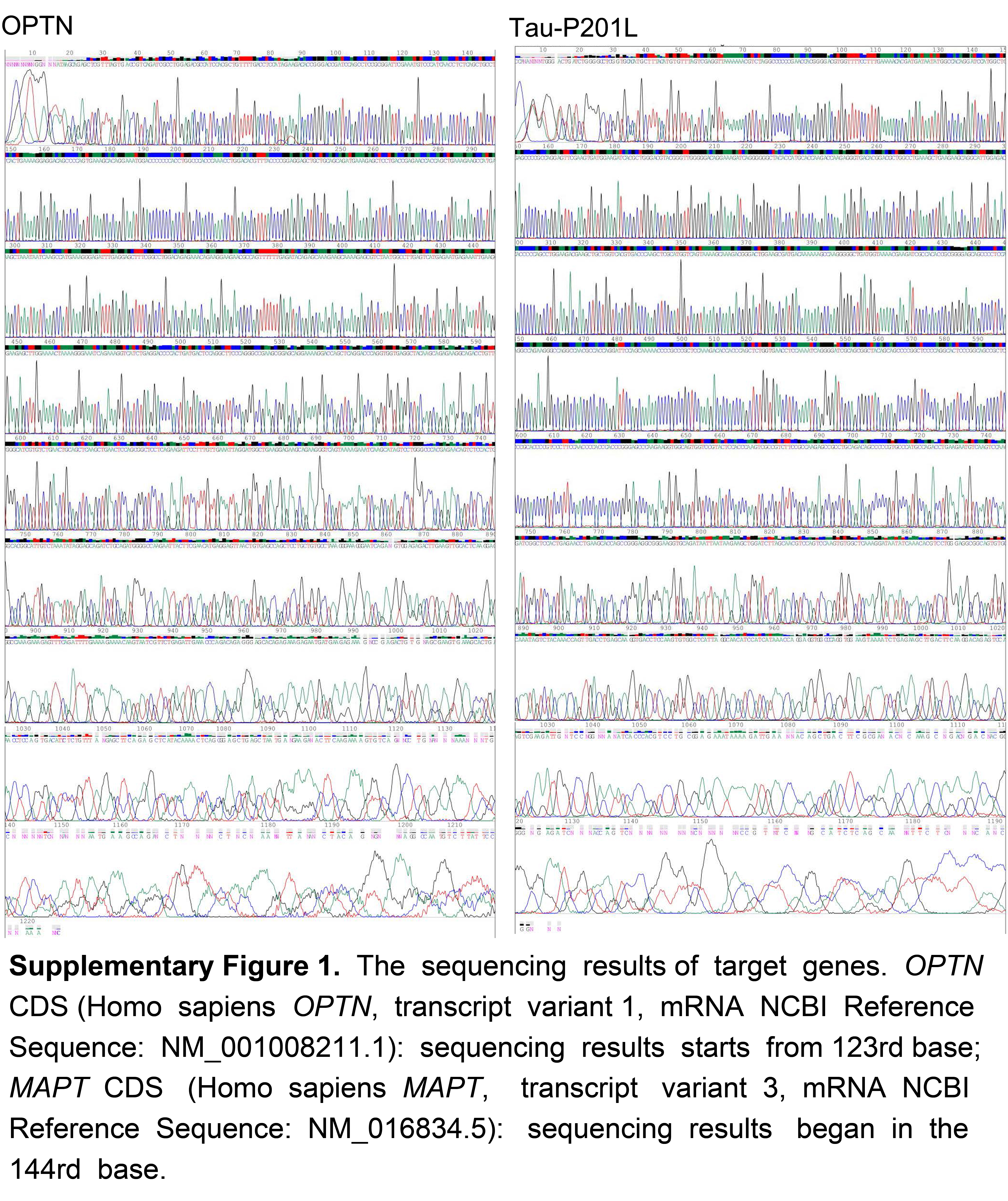

Supplement: Supplementary file 1 — Supplementary Figure 1 [file 41398_2022_2004_MOESM1_ESM.jpg]

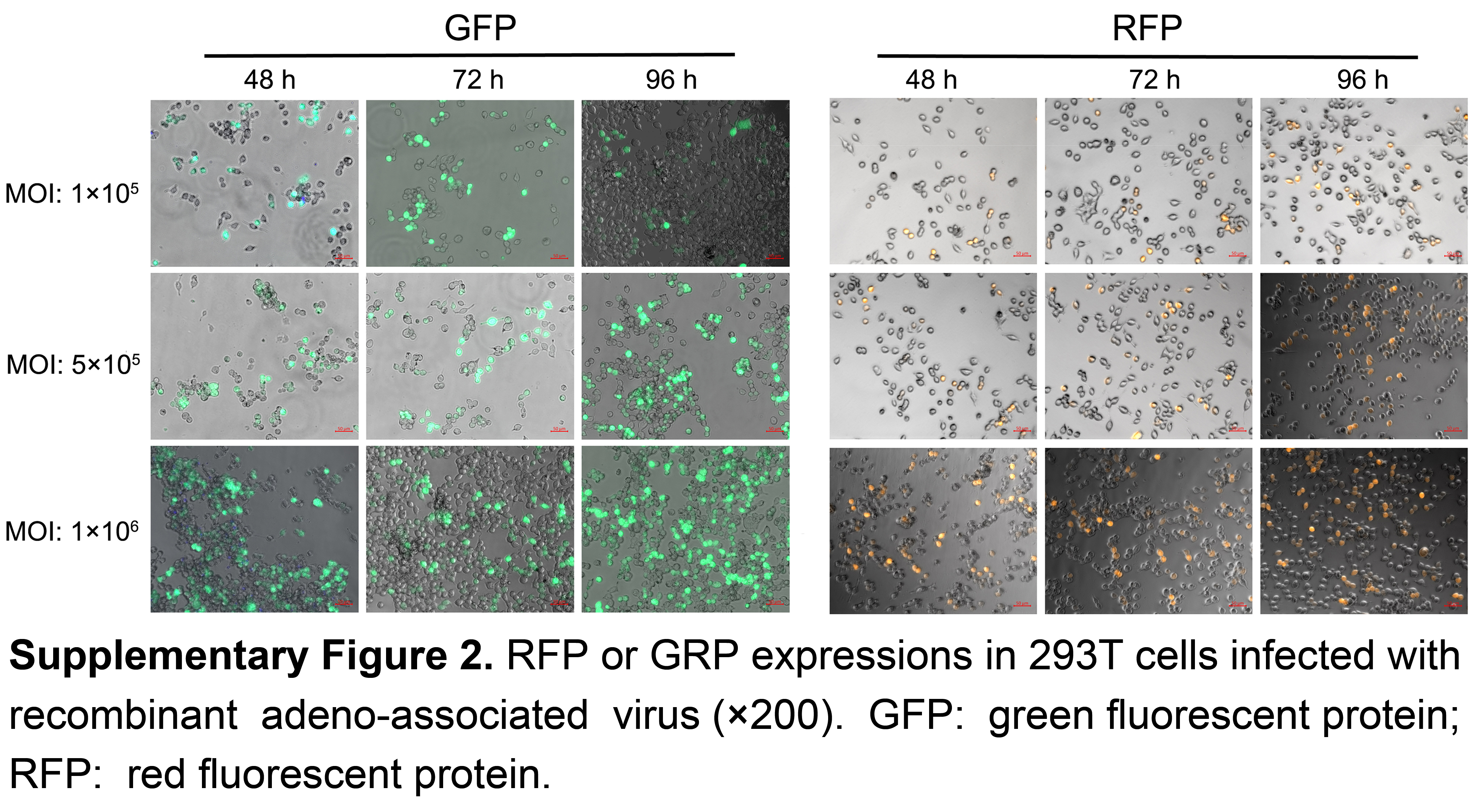

Supplement: Supplementary file 2 — Supplementary Figure 2 [file 41398_2022_2004_MOESM2_ESM.jpg]

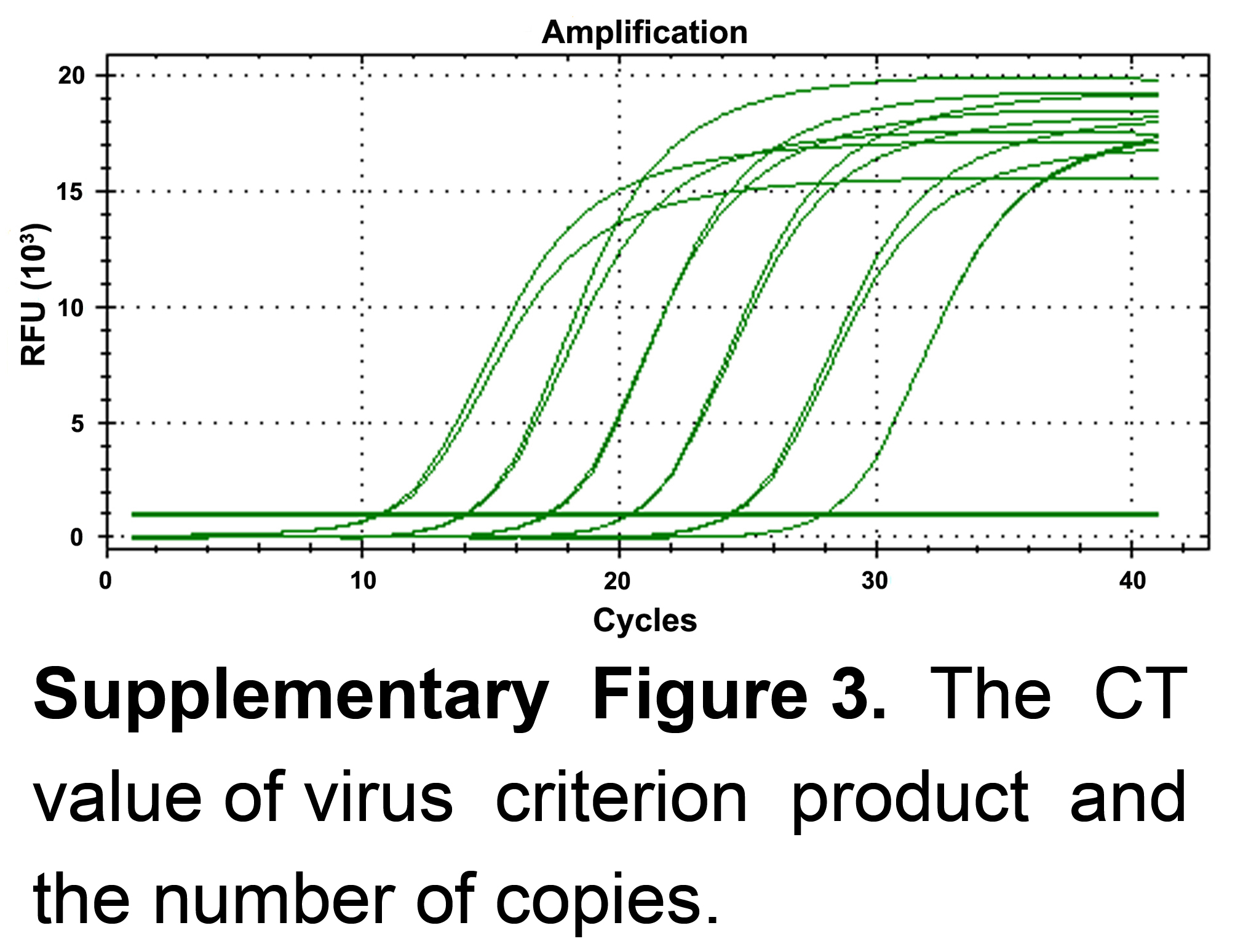

Supplement: Supplementary file 3 — Supplementary Figure 3 [file 41398_2022_2004_MOESM3_ESM.jpg]
