# Supplementary material for: OPTN attenuates the neurotoxicity of abnormal Tau protein by restoring autophagy
Source: Transl Psychiatry. 2022 Jun 4;12:230. doi: 10.1038/s41398-022-02004-x (PMC9167278; doi:10.1038/s41398-022-02004-x)
Supplement: Supplementary file 4 — Supplementary Table 1 [file 41398_2022_2004_MOESM4_ESM.docx]

**Supplementary Table 1. Sequences of the primers for reverse transcription‑quantitative PCR (RT‑qPCR).**

| Gene | Primers (5′-3') |
| --- | --- |
|  |  |
| *Map1lc3a* | Forward: CATGAGCGAGTTGGTCAAGA  Reverse: TTGACTCAGAAGCCGAAGGT |
|  |  |
| *Becn1* | Forward: TGAAATCAATGCTGCCTGGG  Reverse: CCAGAACAGTATAACGGCAACTCC |
|  |  |
| *Casp3* | Forward: ATGGAGAACAACAAAACCTCAGT  Reverse: TTGCTCCCATGTATGGTCTTTAC |
|  |  |
| *Actb* | Forward: GAAATCGTGCGTGACATCAAAG  Reverse: TGTAGTTTCATGGATGCCACAG |
|  |  |
