# Supplementary material for: OPTN attenuates the neurotoxicity of abnormal Tau protein by restoring autophagy
Source: Transl Psychiatry. 2022 Jun 4;12:230. doi: 10.1038/s41398-022-02004-x (PMC9167278; doi:10.1038/s41398-022-02004-x)
Supplement: Supplementary file 5 — Supplementary Table 2 [file 41398_2022_2004_MOESM5_ESM.docx]

**Supplementary Table 2. The viral titres were assayed by real time quantitative PCR (qPCR).**

|  | AAV-OPTN | | AAV-Tau-P301L | |
| --- | --- | --- | --- | --- |
|  |  | |  | |
| E (efficiency for criterio amplification ) | 94.90% | | 94.90% | |
|  |  | |  | |
| Ct1 (criterion) | 13.88 | 13.94 | 13.88 | 13.94 |
|  | 14.00 |  | 14.00 |  |
|  |  | |  | |
| Ct2 (sample) | 16.80 | 16.79 | 16.73 | 16.79 |
|  | 16.77 |  | 16.84 |  |
|  |  | |  | |
| multiple of criterion amplification | 7 | | 7 | |
|  |  | |  | |
| dilution multiple | 1000 | | 1000 | |
|  |  | |  | |
| R (ratio) | 0.15 | | 0.15 | |
|  |  | |  | |
| copies (criterion) | 1.0×10^7^ | | 1.0×10^7^ | |
|  |  | |  | |
| viral titre (vg/ml) | 1.5×10^12^ | | 1.5×10^12^ | |
|  |  | |  | |
